# Effect of Graphene Oxide Nano-Sheets on Structural, Morphological and Photocatalytic Activity of BiFeO_3_-Based Nanostructures

**DOI:** 10.3390/nano9091337

**Published:** 2019-09-19

**Authors:** Syed Irfan, Guang-xing Liang, Fu Li, Yue-xing Chen, Syed Rizwan, Jingcheng Jin, Zheng Zhuanghao, Fan Ping

**Affiliations:** 1 Shenzhen Key Laboratory of Advanced Thin Films and Applications, College of Physics and Optoelectronic Engineering, Shenzhen University, Shenzhen 518060, China; syedirfan@szu.edu.cn (S.I.); lgx@szu.edu.cn (G.-x.L.); lifu@szu.edu.cn (F.L.); chenyx@szu.edu.cn (Y.-x.C.); jingchengjin@szu.edu.cn (J.J.); fanping@szu.edu.cn (F.P.); 2 Key Laboratory of Optoelectronic Devices and Systems of Ministry of Education and Guangdong Province, College of Optoelectronic Engineering, Shenzhen University, Shenzhen 518060, China; 3 Physics Characterization and Simulations Lab (PCSL), School of Natural Sciences (SNS), National University of Sciences and Technology (NUST), Islamabad 44000, Pakistan; syedrizwanh83@gmail.com

**Keywords:** sol-gel, graphene oxide, nanohybrids, band-gap, water-treatment

## Abstract

Photocatalysts are widely used for the elimination of organic contaminants from waste-water and H_2_ evaluation by water-splitting. Herein, the nanohybrids of lanthanum (La) and selenium (Se) co-doped bismuth ferrites with graphene oxide were synthesized. A structural analysis from X-ray diffraction confirmed the transition of phases from rhombohedral to the distorted orthorhombic. Scanning electron microscopy (SEM) revealed that the graphene nano-sheets homogenously covered La–Se co-doped bismuth ferrites nanoparticles, particularly the (Bi_0.92_La_0.08_Fe_0.50_Se_0.50_O_3_–graphene oxide) LBFSe50-G sample. Moreover, the band-gap nanohybrids of La–Se co-doped bismuth ferrites were estimated from diffuse reflectance spectra (DRS), which showed a variation from 1.84 to 2.09 eV, because the lowering of the band-gap can enhance photocatalytic degradation efficiency. Additionally, the photo-degradation efficiencies increased after the incorporation of graphene nano-sheets onto the La–Se co-doped bismuth ferrite. The maximum degradation efficiency of the LBFSe50-G sample was up to 80%, which may have been due to reduced band-gap and availability of enhanced surface area for incoming photons at the surface of the photocatalyst. Furthermore, photoluminescence spectra confirmed that the graphene oxide provided more electron-capturing sites, which decreased the recombination time of the photo-generated charge carriers. Thus, we can propose that the use of nanohybrids of La–Se co-doped bismuth ferrite with graphene oxide nano-sheets is a promising approach for both water-treatment and water-splitting, with better efficiencies of BiFeO_3_.

## 1. Introduction

The over-exploitation of resources and environmental pollution are becoming worse with the rapid advancement of global industrialization, which has caused to serious harm to ecological balance. In the recent decades, semiconducting photocatalysts have been the most favorable route for pollution of water and air remediation after photocatalytic oxidation [1,2]. Titanium dioxide is the most reliable photocatalyst due to its strong oxidizing activity, high photo-reactivity, and long-term stability for chemical corrosion and photo-corrosion [3,4]. However, its large band-gap (3.2–3.6) eV is the only hindrance for its practical applicability on a huge scale. Thus, extensive research has already been done to investigate the factors for improving the photocatalytic activity of titanium dioxide by some modifications, such as single or co-doping. Moreover, Poojitha et al. found that the doping of Eu^3+^ and Fe^3+^ into ZnO and Fe^3+^ nano particles and Co and Ni into ZnS nanoparticles significantly improved their diamagnetic and paramagnetic properties. They also found that the Eu^3+^-doped (3%) samples showed an enhanced photocatalytic activity for RhB that degraded 97% within 50 min under UV-light [5,6,7,8].

From the literature, it can be seen that doped titanium dioxide photocatalysts can exhibit some activity under visible-light but can show bad performance and less stability [9,10]. Some researchers have found that carbon-based materials (such as graphene and nanotubes) with titanium dioxide could possibly enhance photocatalytic performance by increasing adsorption wavelength [11,12]. As such, many researchers have been determined to expand the absorption wavelength range of photocatalysts under visible light irradiations. Usually, conduction band levels are low in semiconducting oxides because of the production of deep valence bands with O_2_p, which constrains the evolution of stable photocatalysts under visible light. As such, the orbitals of foreign impurities may help to control the valence band rather than O_2_p.

Bismuth is supposed to be a promising element to control valence bands, and Bi-based perovskite oxides showed a higher photocatalytic activity for visible-light [13]. The perovskite bismuth ferrite has received great attention for its optical applications, particularly photocatalytic activity, owing to its chemical stability and low band-gap (2.2 eV). Various attempts have been made to make BiFeO_3_ (BFO) a more efficient visible-light photocatalyst, such as that by Li et al., who found that the sub-microcubes of BFO exhibited better photocatalytic performance compared to microcubes and microspheres under visible-light [14]. Moreover, the nanostructures of SrTiO_3_-coated BFO demonstrated an enhanced photocatalytic performance for the production of H_2_ for visible-light illumination [15]. Many fabrication techniques have been studied for the synthesis of BFO nanostructures, such as the tartaric acid-assisted gel strategy, [16] the polymer-directed solvothermal route, [17] the ferrioxalate precursor method, [18] combustion synthesis, [19,20] hydrothermal synthesis [21,22] microwave-induced solid-state decomposition, [23] co-precipitation, [24,25] the molten-salt method, [26,27] the hydrothermal method assisted with polymer, [28] the Pechini and modified Pechini methods, [29,30] microwave–hydrothermal synthesis, [31] the mineralizer-assisted hydrothermal technique, [32] the polymeric precursor method, [33,34] sol–gel, [35,36] micro-emulsion techniques, [37] the mechanochemical approach, [38] and the ethylene diamine tetra acetic acid (EDTA) complexing sol–gel method [39].

In photocatalysis, the oxidation process mainly depends upon the rate of recombination of photo-generated charge carriers. As such, photocatalytic performance reduces if the recombination rate increases [40]. Therefore, it is needed to synthesize a BFO-based structure that provides photo-generated charge carrier-capturing sites. From the literature, it can be seen that graphene and its oxides with BFO, such as nanohybrids, could help in enhancing photocatalytic performance by capturing photo-generated charge carriers which delay the recombination time of electrons and holes [41,42]. Zhuoxuan et al. reported that the nanohybrids of BFO with graphene showed a higher photocatalytic performance for visible-light [43]. Carbon-based materials showed tremendous properties, such as graphene-based materials which exhibit high charge movements and electron conductivity. Li et al. fabricated 3D-BFO nanoparticles embedded into various graphene oxide concentrations by a hydrothermal method. They found that the submicron cubes of a BFO composite with 3 mg/mL graphene oxide exhibited a 92% degradation efficiency for methylene blue (organic dye) in 140 min. This may be attributed to an enlarged surface area compared to the pure BFO and 3D morphology [44]. The graphene was composed of single layer carbon atoms that were hexagonally arranged. From the literature, it has been established that graphene shows good photocatalytic activity compared with pure BFO [45].

In another study, the nano-composites of BFO with reduced graphene oxide were synthesized by chemical route. These nano-composites successfully degraded Rhodamine B dye up to 94% under visible-light within 2 h. It was also found that reduced graphene oxide (rGO)-BFO composite exhibited a tremendous photoelectrochemical performance for the splitting of water, which may be due to the availability of free charge carriers, increasing the recombination time that effectively enhanced photocatalytic activity [46]. Pai et al. reported that composites of BFO with nitrogen-doped graphene significantly improved the photo-degradation efficiencies of BFO by degrading Congo Red as an organic pollutant [47]. It is well-known that photocatalyst-produced strong oxidizing agents create electronic holes in water by the irradiation of light (ultraviolet or visible) [48]. These hydroxyl radicals oxidize organic contaminants into less harmful compounds. Usually, the general photocatalytic performance of a semiconductor photocatalyst is based on several aspects. The most important is the effective splitting of photo-generated charge carriers (e^‒^–h^+^) pairs which refine photocatalytic performance [49]. Basically, photocatalysis are followed by a fundamental mechanism which starts from the production of e^‒^–h^+^ pairs in semiconductor material. The e^‒^–h^+^ pairs are transported to the surface of a photocatalyst to create reducing species, and the surface defects act as trapping sites for electrons and then detach to produce super oxide anion radicals. Simultaneously, the holes react with H_2_O molecules or with hydroxyl (OH) groups to yield hydroxyl radicals, which destroy the organic contaminants of waste-water.

The photocatalytic efficiency of a decent semiconductor photocatalyst is intensely based on its photo-generated charge carriers’ separation and band-gap, as well as the morphology of the photocatalyst [50]. The band-gap of BFO can be tuned by inducing some dopants into various transition metals and non-metals, and its surface morphology can be changed by making composites with graphene oxide. However, no study has been reported the co-doping of selenium and lanthanum into BFO nanohybrids with graphene oxide. Here, La^3+^ and Se^+4^ co-doped BFO nanoparticles, fabricated by a double solvent sol-gel method, and the nanohybrid structures with graphene oxide were fabricated by a co-precipitation method. The photocatalytic activity for La^3+^ and Se^+4^ co-doped BFO with their nanohybrid with graphene oxide was thoroughly studied. It has been pointed out that dye shakes its adsorption on the co-doped and hybrid composites and degrades organic contaminants. With this, we have tried to explain the mechanism of organic dyes in a broad-range spectrum in the presence of co-doped BFO-graphene oxide. The presented results can help in understanding the co-doped BFO-graphene oxide nanohybrids.

## 2. Materials and Methods

### 2.1. La^3+^ and Se^+4^ Co-Doped Nanoparticles

The La^3+^ and Se^+4^ co-doped BFO with Bi_1-x_La_x_Fe_1-y_Se_y_O_3_, (*x =* 0.08, *y =* 0.0, 0.10, 0.25, 0.50, and 1) were fabricated by the double solvent sol–gel method. The detailed experimental process has been discussed in other report [50,51].

### 2.2. Synthesis of BFO/Graphene Oxide Nanohybrids

The nanohybrids of La^3+^ and Se^+4^ co-doped BFO with graphene oxide (1 mg/mL) were fabricated by the co-precipitation method, abbreviated as (Bi_0.92_La_0.08_FeO_3_–graphene oxide, as LBF–G), (Bi_0.92_La_0.08_Fe_0.90_Se_0.10_O_3_–graphene oxide, as LBFSe10–G), (Bi_0.92_La_0.08_Fe_0.75_Se_0.25_O_3_–graphene oxide, as LBFSe25–G), (Bi_0.92_La_0.08_Fe_0.50_Se_0.50_O_3_–graphene oxide, as LBFSe50–G), and (Bi_0.92_La_0.08_Fe_0.0_Se_0.100_O_3_–graphene oxide, as LBFSe100–G). In this synthesis approach, we prepared a solution of 10 mL of acetic acid (Sigma Aldrich, Shenzhen, China) and 10 mL of ethylene glycol (Sigma Aldrich, Shenzhen, China), and we both mixed together before stirring for 60 min at room temperature. After that, 0.04 molar of La–Se co-doped BFO nanoparticles were added, and the solution was sonicated for 3 h at 60 °C (Solution A). Meanwhile, the dispersion of graphene oxide (1 mg/1 mL) was also prepared in deionized water (Solution B). Both the solutions (Solution A + Solution B = Solution C) were mixed together, sonicated for 10 min, and then placed for magnetic stirring for 1 h at 80 °C. After 1 h, Solution C was cooled down at room temperature, and its precipitates were found. These precipitates were washed with deionized water several times to maintain pH ~1. The resulting filtrate was dried in a common oven for 1 day to obtain nanohybrids.

### 2.3. Characterizations

The crystal structure analysis of the nanohybrids was executed by an X-ray diffract meter (XRD, Bruker, Billerica, Massachusetts, USA), within the range of 2θ = 20°~80° using Cu-Kα (*λ* = 0.15418 nm) radiation (scan speed of 2°/min). Field emission electron microscopy (FESEM, Hitachi-S5500, Berlin, Germany) was used to investigate morphological behavior. X-ray photoelectron spectroscopy (XPS; Thermo Scientific Escalab-250 xi equipped with monochromatic AlKα (Netherlands, Europe) was engaged to analyze the elemental composition and chemical state. The photoluminescence spectrum (Hitachi luminescence spectrometer (F-4500), Berlin, Germany was observed and provided the recombination rate of the photo-generated charge carriers. The photocatalytic performance of the nanohybrids were observed by UV-vis absorption spectroscopy (PerkinElmer, (Akron, OH, USA), Lambda 950 photo spectrometer system).

## 3. Results and Discussion

### 3.1. Crystal Structure Measurement

**Figure 1:**
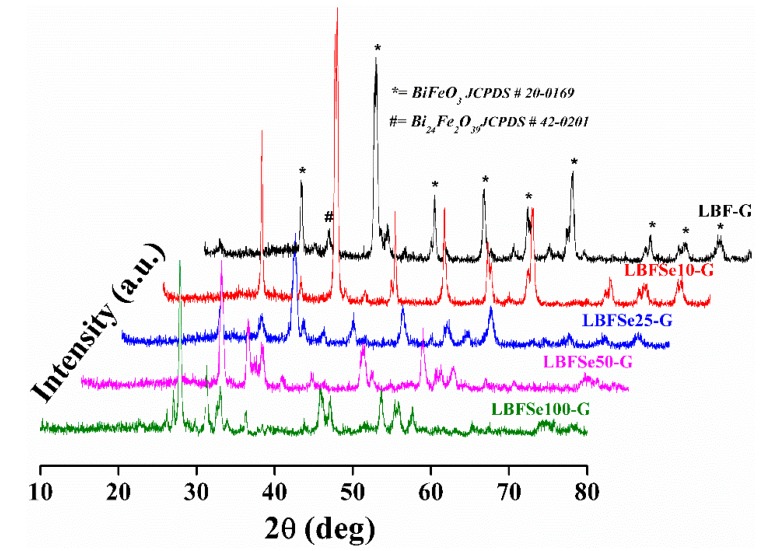
The XRD pattern of La^3+^ and Se^+4^ co-doped BiFeO_3_ (BFO) nanohybrids with graphene oxide samples.

The well crystalline XRD patterns of nanohybrids, LBF–G, LBFSe10–G, LBFSe25–G, LBFSe50–G, LBFSe100–G are demonstrated in Figure 1. We observed that the diffracted peaks of the nanohybrids sample corresponded to the phase transition from the rhombohedral to the distorted orthorhombic structure with minor impurity peaks. The diffracted pattern of nanohybrids matched with Joint Committee on Powder Diffraction Standards (JCPDS) card no. 20-0169 and 42-0201 without presence of unwanted impurity phases. 

**Figure 2:**
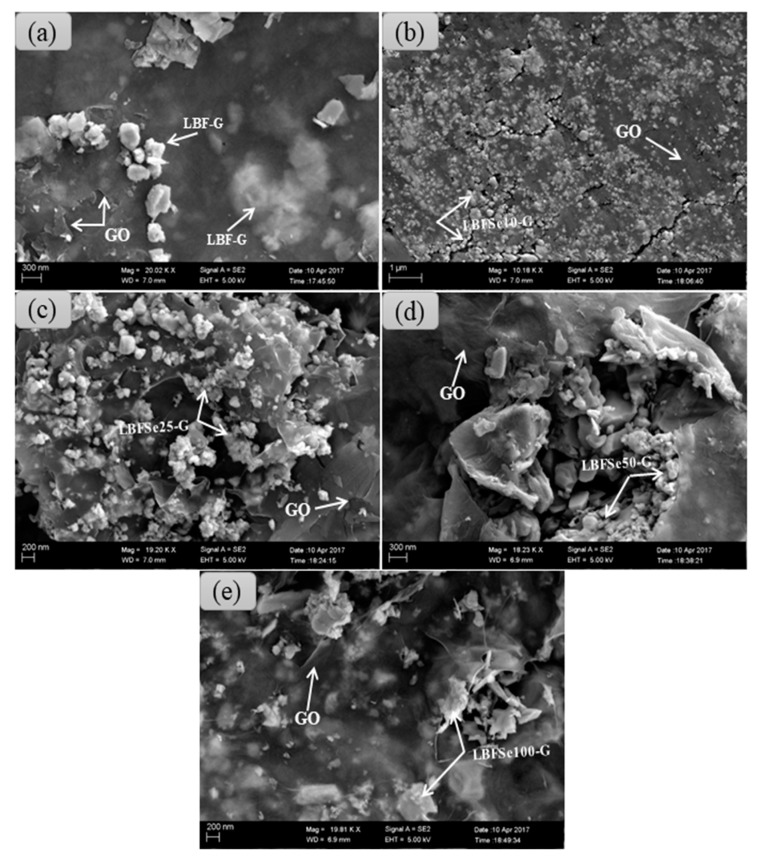
SEM micrographs of (**a**) Bi_0.92_La_0.08_FeO_3_–graphene oxide (LBF–G), (**b**) Bi_0.92_La_0.08_Fe_0.90_Se_0.10_O_3_–graphene oxide (LBFSe10–G), (**c**) Bi_0.92_La_0.08_Fe_0.75_Se_0.25_O_3_–graphene oxide (LBFSe25–G), (**d**) Bi_0.92_La_0.08_Fe_0.50_Se_0.50_O_3_–graphene oxide (LBFSe50–G), and (**e**) Bi_0.92_La_0.08_Fe_0.50_Se_0.50_O_3_–graphene oxide (LBFSe100–G).

However, a remarkable increase in the intensity of the peak lying at an angle ~27.50˚ corresponding to Bi_24_Fe_2_O_39_ was observed in nanohybrids with increase in the Se-concentration from 0% to 100%. The prominent peaks (012), (104) and (110) of the rhombohedral structure were diminished with the increasing Se concentration in nanohybrids. The calculated lattice parameters of nanohybrid samples were a = 5.593 nm ± 0.016 and b = 13.912nm ± 0.033 and the average crystallite sizes of nanohybrids were 17, 22, 18, 24, and 27 nm for LBF–G, LBFSe10–G, LBFSe25–G, LBFSe50–G, and LBFSe100–G, respectively. The morphology of the as-synthesized nanohybrids was studied by field emission scanning electron microscopy, as presented in Figure 2. The micrographs of nanohybrids showed that the graphene oxide flakes were not covered properly onto the entire surface of La-doped BFO, but graphene oxide nano-sheets preferably accumulated with each other relative to getting covered the surface by nanoparticles. We observed that the surfaces of LBFSe10–G and LBFSe25–G nano-sheets were fully decorated with graphene oxide. Cracks were found in the LBFSe10–G sample, which may have degraded its photocatalytic activity, while the major surface of the LBFSe25–G sample was heavily coated with graphene oxide. Interestingly, the micrographs of the LBFSe50–G nanohybrid sample showed that the 50% the selenium doped LBF–G nanoparticles were homogeneously coated by graphene oxide, which may explain how the LBFSe50–G sample was entirely covered by the crumpled sheets of graphene oxide producing better photocatalytic activity. The LBFSe100–G sample was irregularly covered by graphene oxide, which resulted the intermediate transformation between nano-sheets and nanoparticles, as observed at 100% doped Se^+4^ onto La^+3^ doped-BFO.

### 3.2. Electronic Properties

**Figure 3:**
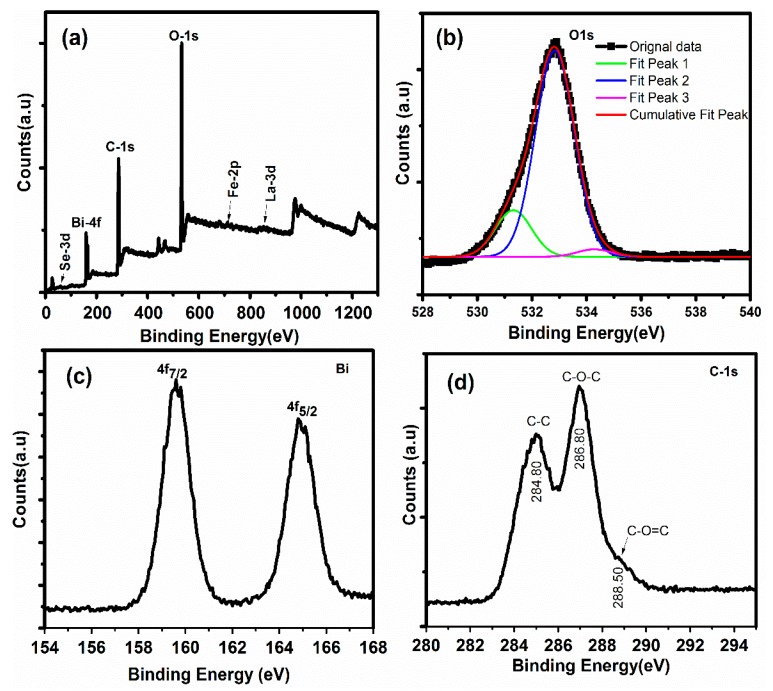
X-ray photoelectron spectroscopy (XPS) spectra of LBFSe50–G: (**a**) Survey spectra, (**b**) oxygen-1s, (**c**) Bismuth-4f, and (**d**) carbon-1s.

XPS was used to examine the electronic states of species in each sample for the entire series of samples. The XPS results of the highly efficient LBFSe50–G nanohybrid are presented in Figure 3. From XPS measurements, it could be seen that the metallic species of constitutes was not present. The presence of Se-3d, Bi-4f, C-1s, O-1s, Fe-2p and La-3d constituents was confirmed without any extra impurities, as displayed in Figure 3a. The asymmetric O-1S spectrum was de-convoluted into three parts. The peak positioned at 532.81 eV corresponded to the O-1s spectrum and covered a major part of spectrum, as reported in the literature [52] and assigned to bonding with cationic species.

The binding energy of O-1s in the range of 530.20–532.30 eV and 533.20–534.80 eV was allocated to oxygen vacancies and the oxygen that adsorbed on the surface or the hydroxyl ion groups, respectively [53], as depicted in Figure 3c, which displays the doublet located binding energies at 159.20 and 164.80 eV correspond to the core lines of Bi-4_f5/2_ and Bi-4_f7/2_, respectively, and were assigned to the Bi^+3^ electronic state as reported earlier [54]. There were two major peaks of C-1s spectra, as shown in Figure 3d. The first peak positioned at 284.80 eV showed the presence of C–C bond, and the second peak positioned at 286.80 eV showed the C–O–C bond coordination. The first peak may have been due to carbon tape, while the second peak reflected the presence of graphene oxide (i.e., C–O–C) [52].

**Figure 4:**
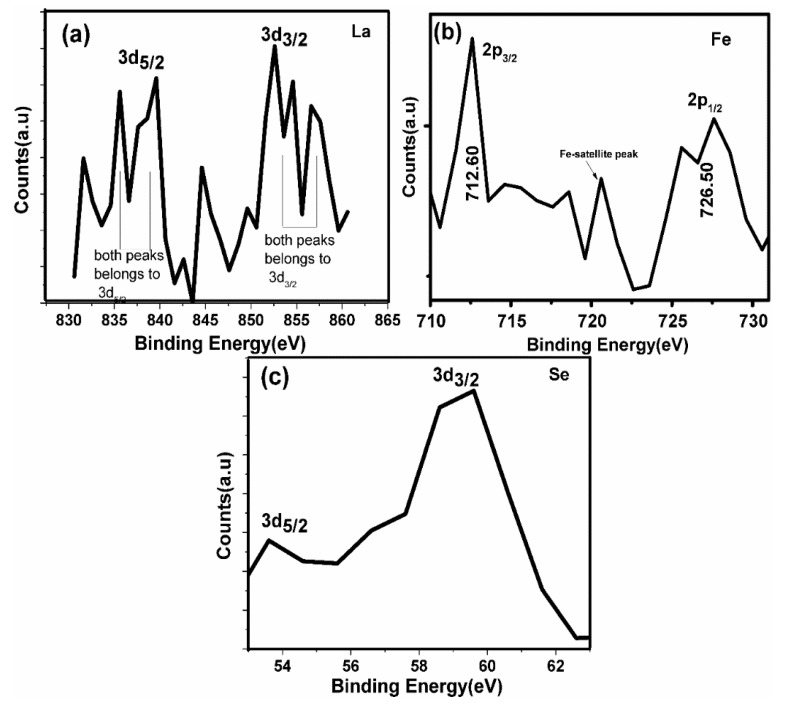
XPS high resolution spectra of the LBFSe50–G system extracted from survey scan (**a**) La-3d (**b**) Fe-2p and (**c**) Se-3d.

No extra bonding of carbon, e.g., with Fe, which could create the possible formation of FeC (iron carbide), was absent from the high resolution C-1s spectra. The XPS spectra (with a high resolution) of the LBFSe50–G sample that had La-3d, Fe-2p and Se-3d have been shown in Figure 4a–c. The XPS spectra of La-3d were similar to that for La_2_O_3_ (Figure 4a) [55]. They contained two strong photoelectron peaks, and their analogous shake-up peaks were positioned a few electron volts higher in binding energies. The photoelectron peaks conforming to La-3d_3/2_ and La-3d_3/2_ were observed at around 853.70 and 836.90 eV, with a spin-orbit splitting of 16.80 eV and shake-up lines situated at 840.2 and 857.3 eV, respectively [56]. The Fe oxidation states are shown in Figure 4b.

The Fe-2p_3/2_ and Fe-2p_1/2_ peaks of Fe^+3^ were located at binding energies 712.60 and 726.50 eV, respectively, obtained from the spin–orbit interaction. No obvious peak was observed for Fe^+2^ near 709.50 eV. The presence Fe^+3^ also confirmed the existence of the Fe-2p3/2 peak at 720.60 eV [57]. The core level Se-3d_3/2_ and Se-3d_5/2_ doublet peaks arose at binding energies of 53.80 and 59.0 eV, respectively, which were attributed to the Se presence in the +4 oxidation state. The XPS core spectra of the Se-3d peak in its oxide form showed a doublet. The XPS spectra of Se-3d in pure Se (i.e., metallic) showed the absence of spin orbit splitting [58]. The lower binding energy peak (53.80 eV) may have been attributed to Se impurity, and the higher binding energy peak (59.90eV) could be due to the Se-3d peak in the SeO_2_ [59].

### 3.3. Diffuse Reflectance Spectrum

**Figure 5:**
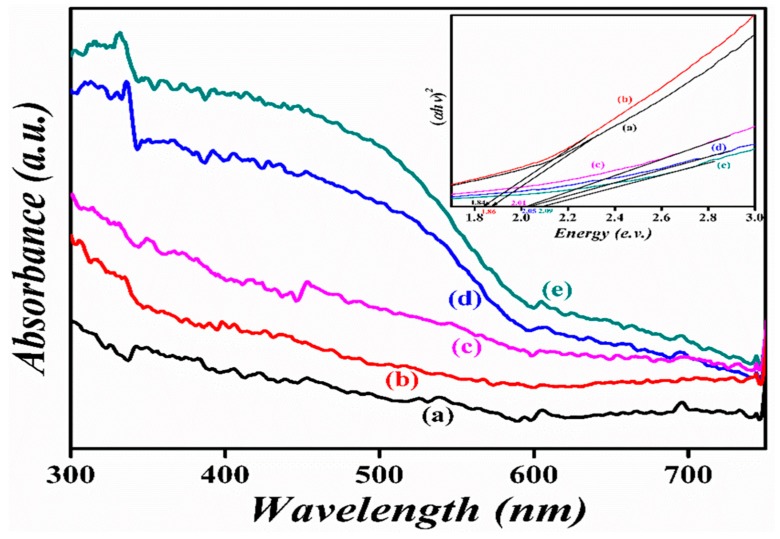
The diffuse reflectance spectra of (a) LBF–G, (b) LBFSe10–G, (c) LBFSe25–G, (d) LBFSe50–G, and (e) LBFSe100–G.

The UV-vis diffuse reflectance spectra of the pristine BFO and nanohybrids of La^3+^ and Se^+4^ co-doped BFO with graphene oxide samples were examined, as presented in Figure 5. The band-gap estimated values were 1.86, 1.84, 2.09, 2.05, and 2.01 eV for LBF–G, LBFSe10–G, LBFSe25–G, LBFSe50–G, and LBFSe100–G, respectively. In common, the band-gap of semiconductors usually depends on crystallite size, alteration in lattice parameters, surface morphology, and dopants. However, if the crystallite size is higher than the critical size required for BFO, then it could not affect the band-gap anymore [60].

### 3.4. Photoluminescence Spectroscopy

**Figure 6:**
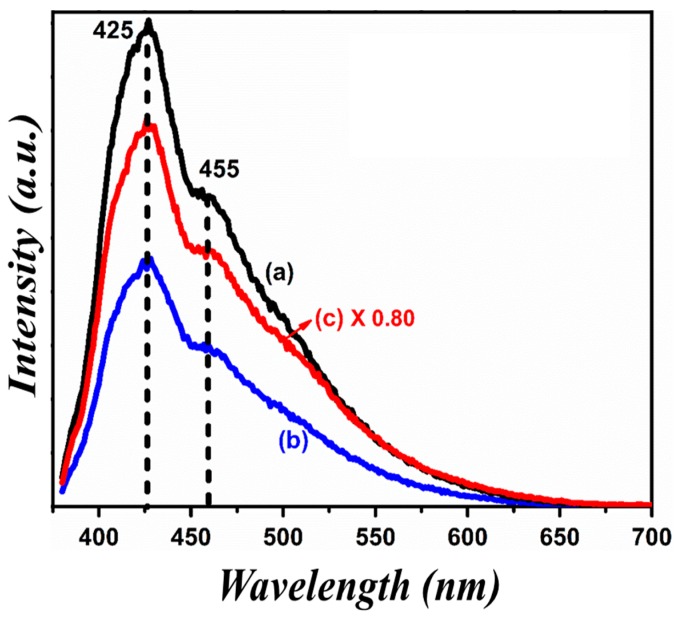
Photoluminescence spectra of (a) LBFSe10–G, (b) LBFSe50–G, and (c) LBFSe100–G.

Photoluminescence emission (λ_ex_ = 300 nm) spectrum was accomplished to observe the recombination rate of the photo-generated charge carriers of the as-synthesized nanohybrids of La^3+^ and Se^+4^ co-doped BFO with graphene oxide. The photocatalytic performance of semiconductor photocatalysts mainly depends on the recombination time of photo-generated charge carriers [61,62,63]. Figure 6 indicates the photoluminescence emission spectra of La^3+^ and Se^+4^ co-doped BFO with graphene oxide nanohybrids series. Asymmetric broad spectra in the range of 380–700 nm with prominent peaks identified in the positions of 425 and 455 nm were observed. Usually the band-gap of pristine BFO lies in green visible region; the emission peak at 455 nm corresponds to the transition of electrons from the valence band to conduction band [64]. In our case, no variation in band-gap after graphene oxide coating was observed because photoluminescence emission is a surface phenomenon and not a bulk phenomenon. The calculated band-gap lied at position 455 nm (2.72) in the nanohybrid samples. The higher values of selenium concentration in nanohybrid samples changed their photoluminescence emission intensity of spectra. Moreover, the lowest value of photoluminescence emission intensity observed in curve (b) corresponded to the LBFSe50–G sample, as compared to curve (a) and (c) for BFO and LBFSe100–G, respectively.

This lowest value of photoluminescence emission spectra corresponded to the lower charge recombination rate of the photo-generated charge carriers that increased the photocatalytic performance. From photoluminescence emission data, we could predict that the LBFSe50–G sample may give a maximum value of photo-degradation.

A very small amount of oxygen defects were found in XPS data but were not found in photoluminescence emission data; usually, the band centered at 530–540 nm was attributed to the existence of oxygen vacancies [65].

### 3.5. Photocatalytic Performance

**Figure 7:**
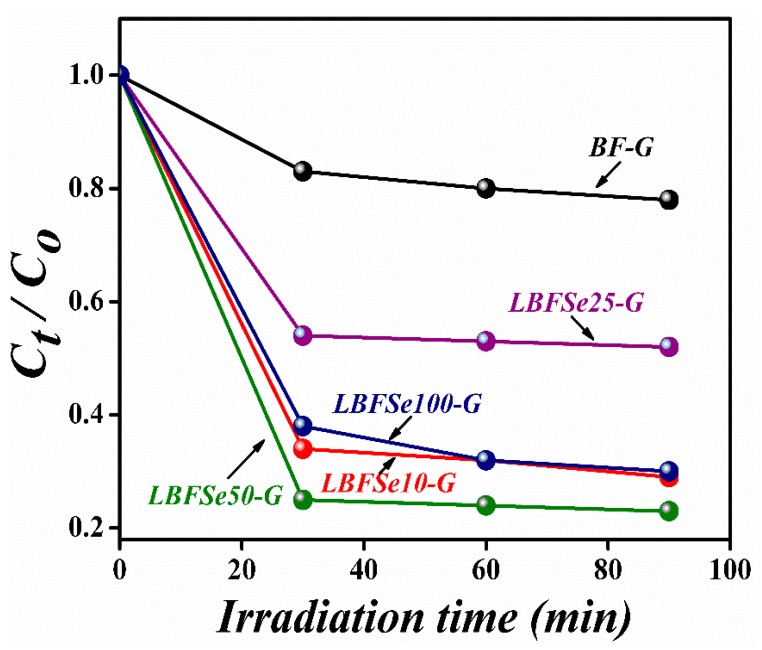
The photo-degradation efficiencies of Congo Red (CR) in the presence of, (a) BFO–G, (b) LBFSe10–G, (c) LBFSe25–G, (d) LBFSe50–G, and (e) LBFSe100–G.

The photocatalytic performance of the nanohybrids of La^3+^ and Se^+4^ co-doped BFO with graphene oxide was performed by the elimination of the organic pollutant Congo Red (CR) under visible-light obtained from a Xenon lamp (power = 300 W and filter wavelength = 420 nm) [66,67]. The concentration of the CR organic dye was 100 mg/L in deionized water. A 10 mg amount of photocatalyst was added into the CR solution and then stirred in the dark for 2 h to get a homogenous solution. After that, the solution was exposed to visible-light with a 3 m distance from the light source that had an intensity of 132 mW/cm^2^. The origin of the enhanced photocatalytic performance in BFO is its low recombination rate and enlarged surface area. As the surface area of the photocatalyst increases, the dye gains direct contact with the catalyst, making redox reactions easy to occur. By increasing Se-doping, the average distance between trap sites decreases, causing a large recombination rate [68,69,70]. However, the presence of graphene oxide nano-sheets provides extra trapping sites for incoming photo-generated charge carriers, which results in better photocatalytic activity under visible-light. From Figure 7, it can be seen that for the pristine BFO photocatalyst, almost 20% of CR was degraded after the exposure to visible irradiation for 90 min.

By using the LBF–G nanohybrid as a photocatalyst, over 72% of CR was removed within the same length of irradiation time (90 min). For the LBFSe25–G nanohybrid sample, almost 50% of CR was degraded. Moreover, with varying the concentrations of selenium onto LBF–G (up to 50%), the degradation efficiency reached 79%. However, the LBFSe100–G nanohybrid sample showed 68% degradation efficiency for CR under visible-light irradiation, which suggests that optimal concentrations of co-dopant and graphene oxide significantly enhanced the photocatalytic activity of BFO. The data suggest that the presence of graphene oxide significantly affects the photocatalytic behavior of La^3+^ and Se^+4^ co-doped BFO nanostructures, as reported in the literature [50]. In literature, it has been reported that the good electron mobility of reduced graphene oxide assists in decreasing the rate of recombination of photo-generated charge carriers and increases the value of photocatalysis. However, in graphene oxide, there are some defect sites due to the existence of epoxide, carboxylic acids, and phenoxide on its surface. As such, graphene oxide behaves like electrical insulator. In our hybrid sample, the reduction in photocatalytic performance was related to the pure samples, and the graphene oxide was converted to reduced-graphene oxide to restore its electrical properties, which was not good enough for charge transfers and electron traps [71,72].

**Figure 8:**
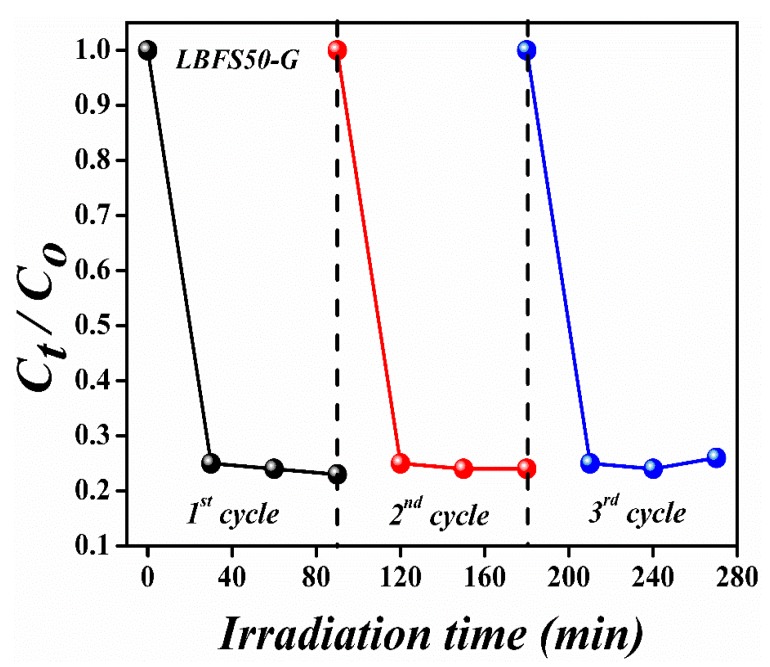
The re-cyclic graph for LBFSe50–G after three cyclic runs under visible-light.

These calculations show that the reduction of graphene oxide to rGO was very low. The low reduction meant the inefficient charge transfer and transportation of photo-generated charges. This inefficient charge transfer and charge trap increased the rate of recombination of charges and caused a decrease in photocatalytic activity. In these samples, the overall degradation was due to adsorption. The band-gap was decreased slightly after adding selenium onto La-doped BFO; however, after incorporation, the band-gap varied significantly from 1.84 to 2.09 eV. The band-gap value for the LBFSe50–G sample was 2.01 eV. The possible photocatalytic mechanism could be explained as: When the visible-light fell on the surface of the photocatalyst, the electron and holes were created. The electron-hole pair further produced H_2_O and CO_2_ as by-products after reacting with the hazardous organic compound. The mechanism started with the absorption of photons, then led to the creation of electrons and hole pairs, and, finally, ended with the production of superoxide radicals for the degradation of organic pollutant. The highest photocatalytic degradation efficiency of LBFSe50–G might have been due to the availability of extra electron-capturing centers due to the availability of the larger surface areas of graphene nano-sheets. These captured electrons converted O_2_ into O_2_^•^, where the H_2_O molecules were converted into OH^•^. Both radical species efficiently degraded the organic pollutant and converted it into a harmless product (CO_2_ and H_2_O). The stability of a photocatalyst is also important parameter for its industrial applications. As such, the chemical stability of the LBFSe50–G sample was observed its after repeating the photocatalytic experiment three times, as shown in Figure 8. It was observed that the LBFSe50–G sample showed nearly the same efficiency, which confirmed that it can be used several times for the degradation of organic pollutant by visible-light photocatalysis.

## 4. Conclusions

The nanohybrids of La^3+^ and Se^+4^ co-doped BFO with graphene oxide were synthesized by the co-precipitation method. A structural analysis from X-ray diffraction confirmed the transition from the rhombohedral phase to the distorted orthorhombic phase. Moreover, the presence of Se-3d, Bi-4f, C-1s, O-1s, Fe-2p and La-3d constituents was also confirmed from the XPS examination. SEM micrographs revealed that the graphene oxide nano-sheets thoroughly covered the nanoparticles, which provided a broad surface area for the incoming photons of irradiated visible-light. The as -synthesized nanohybrids showed enhanced photocatalytic activity for CR under visible-light, where the LBFSe50–G sample exhibited the highest photocatalytic performance—up to 80%. This may have been due to the decrease of the recombination time for photo-generated charge carriers due to the availability of trapping sites provided by graphene oxide nano-sheets. Therefore, the results suggest that the nanohybrids of La^3+^ and Se^+4^ co-doped with graphene oxide nano-sheets can be promising photocatalysts for the removal of waste-water from industrial waste.
